# The correlation between poor prognosis and increased yes-associated protein 1 expression in keratin 19 expressing hepatocellular carcinomas and cholangiocarcinomas

**DOI:** 10.1186/s12885-017-3431-1

**Published:** 2017-06-23

**Authors:** KyuHo Lee, Kyoung-Bun Lee, Hae Yoen Jung, Nam-Joon Yi, Kwang-Woong Lee, Kyung-Suk Suh, Ja-June Jang

**Affiliations:** 1 Department of Pathology, Seoul National University College of Medicine, 101 Daehak-ro, Jongno-gu, Seoul, 110-744 South Korea; 2 Department of Surgery, Seoul National University College of Medicine, Seoul, 110-744 South Korea

**Keywords:** YAP1, Hepatocellular carcinoma, Cholangiocarcinoma, Combined hepatocellular and cholangiocarcinoma, Progenitor cell

## Abstract

**Background:**

The Hippo pathway plays a vital role in liver regeneration and development by determining cellular lineage and regulating cell proliferation and apoptosis. In this study, we aimed to assess the role of the Hippo pathway in hepatic carcinogenesis and morphogenesis by examining Yes-associated protein 1 (YAP1) expression in the spectrum of hepatic carcinomas based on cellular lineage.

**Methods:**

We examined 913 primary hepatic carcinomas, including hepatocellular carcinomas (HCCs), combined hepatocellular and cholangiocarcinomas (cHC-CCAs), intrahepatic cholangiocarcinomas (IHCCAs) and perihilar extrahepatic bile duct carcinomas (EHBCAs). Our study group was categorized into 8 disease groups, based on histological diagnosis and cytokeratin 19 (CK19) expression, and immunohistochemistry was used to detect and compare YAP1 expression levels between the groups. The eight disease groups we identified were: 1) CK19(−) HCC, 2) CK19(−) scirrhous HCC, 3) CK19(+) HCC, 4) stem cell feature of cHC-CCA, 5) classical cHC-CCA, 6) cholangiolocellular IHCCA, 7) non-cholangiolocellular IHCCA, and 8) EHBCA.

**Results:**

Positive rates of YAP1 were the highest in the EHBCA group (21%). CK19(+) HCC and non-cholangiolocellular IHCCA groups also showed high expression levels (10% -11%), while the CK19 (−) HCC, CK19 (−) scirrhous HCC, cHC-CCA, and cholangiolocellular IHCCA groups showed low expression levels, ranging between 0% and 5%. Survival analysis, restricted to pT1 stage HCCs and IHCCAs, showed poor overall survival for YAP1(+) IHCCA patients (39 ± 17 vs. 109 ± 10 months, mean ± SD, log rank *p*-value 0.005). For HCCs, a trend of poor progression-free survival for YAP1(+) HCCs was observed (39 ± 18 vs. 81 ± 5 months, mean ± SD, log rank *p*-value 0.205)

**Conclusions:**

YAP1 activation was more commonly found in CCAs than in pure HCCs. However, a differing pattern of YAP1 expression between cHC-CCAs and CK19(+) HCCs and the poor prognosis of YAP1 positive hepatic carcinomas suggests that YAP1 may have a preferential role in aggressive tumor behavior, rather than in the determination of cellular lineage in hepatic carcinomas.

**Electronic supplementary material:**

The online version of this article (doi:10.1186/s12885-017-3431-1) contains supplementary material, which is available to authorized users.

## Background

The two major histologic types of primary hepatic carcinomas are hepatocellular carcinomas (HCCs) and cholangiocarcinomas (CCAs). HCC is a malignancy with hepatocellular differentiation and CCA is a malignancy with biliary epithelial (cholangiocytic) differentiation. HCCs are the most common type of primary hepatic carcinomas, followed by CCAs. As the difference of histopathogensis, HCCs and CCAs have different clinical behaviors. HCCs can be treated by some loco-regional treatments, such as embolization, radiofrequency ablation, surgical resection, or liver transplantation. However, a high post-treatment recurrence rate remains a challenge for long-term survival [1]. On the other hand, CCAs have been known to have a poorer prognosis than HCCs [2]. Surgical resection is the first treatment of choice for limited stage CCAs, but more advanced stage CCAs are considered to be chemotherapy-resistant [3].

There are some intermediate entities between HCCs and CCAs. These tumors are characterized by their coexisting hepatocytic and cholangiocytic differentiation in the aspect of morphology and associated protein expression. Tumors typically included in this list are classical type combined hepatocellular-cholangiocarcinomas (cHC-CCAs), cHC-CCAs with stem cell features, HCCs expressing stemness markers, and the cholangiolocellular type of intrahepatic CCAs. Classical type cHC-CCAs are typical collision tumors of unequivocal HCC and CCA components. cHC-CCAs with stem cell features are a newly described subgroup, first categorized in 2010 in the 4th edition of the WHO classification of tumors of the digestive system [4]. cHC-CCAs with stem cell features are a collection of hepatic tumors that display morphological and biological evidence for hepatic stem cell or hepatic progenitor cell differentiation. Markers related to stemness are CD133, epithelial cell adhesion molecule (EpCAM), CD56, and cytokeratin 19 (CK19). These markers are normally expressed in the bile ducts in the terminal portal tracts or in the proliferative ductules, frequently identified in injured hepatic parenchyma, and act as a phenotypic marker for hepatic progenitor cells that play a role in liver regeneration and tumorigenesis [5, 6]. cHC-CCA with stem cell feature group has three subcategories: 1) the typical subtype, 2) the intermediate subtype, and 3) the cholangiolocellular subtype [4]. Differential points within these subcategories are morphologic differences, such as the histological pattern of tumor cells, or the distribution of stemness-expressing cells. The cholangiolocellular subtype was first described in the 2000 3rd edition of the WHO classification as a kind of histologic variant of intrahepatic CCAs [7]. The cholangiolocellular pattern has recently come into the spotlight, as intrahepatic CCAs have been classified into two groups, based on their cell of origin in the biliary system [8]. The first group is CCAs arising from the large bile duct with peribiliary glands and the second group is formed by CCAs arising from the canals of Hering, where the hepatic progenitor cells are located. These two groups have different morphological, etiological, clinicopathological, and molecular features [8]. Cholangiolocellular intrahepatic CCAs are considered the most characteristic type of CCAs arising from the canal of Hering. Therefore, cHC-CCAs with stem cell features and cholangiolocellular CCAs are suggested to share similar biological features and clinicopathological behaviors.

HCCs expressing stemness markers are a well-known HCC prognostic subgroup and are characterized by having a poor prognosis and aggressive tumor behavior [2, 9, 10]. This group shows both the typical histological features of HCCs, as well the expression of stemness markers (CD133, EpCAM, or CK19). Because the only major difference between cHC-CCAs with stem cell features and HCCs expressing stemness markers are their morphological features, a gray zone between two groups, based on the opinion of the pathologist in diagnosis, does exist.

YAP1 (Yes-associated protein 1), also known as YAP65, is the mediator of the mammalian Hippo pathway and is associated with development, organogenesis, and postnatal growth [11]. The physiological role of YAP1 in the liver is the commitment of the intrahepatic bile duct to the induction of the cholangiocyte phenotype in the embryonic period and the enlargement of the hepatic volume by expanding progenitor cells, such as oval cells in the adult liver of animal models [12, 13].

In tumorigenesis, YAP1 has been known as an oncogene, due to of its function in promoting cancer cell survival, proliferation, invasion, and migration in several types of malignancies. YAP1 has been associated with poor prognosis in gastric cancer, gallbladder cancer, and hepatocellular carcinoma [14–16]. A meta-analysis on YAP1 expression in various cancers and its prognostic implication reported that upregulation or nuclear expression of YAP1 indicated a worse disease-free survival time and a reduction in the overall survival time (OS) [17]. Recently YAP1 has been reported to play a role as a tumor suppressor during intestinal regeneration in human colorectal cancer [18].

This study aimed to assess the role of the Hippo pathway in hepatic carcinogenesis and morphogenesis by comparing YAP1 expression in hepatic carcinomas, categorized into eight disease groups (including HCCs, CCAs, and intermediate hepatic carcinomas), and analyzing the clinicopathological characteristics of YAP1 expressing tumors in each disease group.

## Methods

### Patient selection and the collection of clinicopathological parameters

**Fig. 1:**
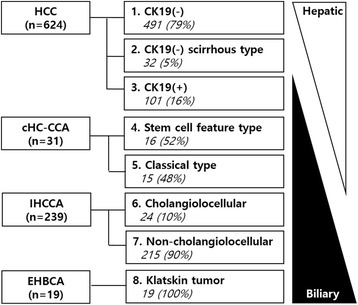
Composition of a total cohort with sequential cellular lineage of hepatic carcinomas. CK19(−) HCC is the most hepatic differentiation and sequentially CK19(−) HCC scirrhous type, CK19(+) HCC, combined hepatocellular-cholangiocarcinoma(stem cell feature, classical type), intrahepatic cholangiocarcinoma (cholangiolocellualr, non-cholangiolocellular) and extrahepatic bile duct carcinoma show biliary differentiation

We studied a total of 913 patients who had been pathologically diagnosed with HCC, cHC-CCA, intrahepatic CCA (IHCCA), and extrahepatic bile duct CCA (EHBCA) from resected specimens, had available medical records, and possessed formalin-fixed paraffin blocks of tumor tissue at the archives of the department of pathology in Seoul National University Hospital stored between 1992 and 2012. A total of 624 HCC patients were included, while 31 cHC-CCA patients were enrolled. A total of 16 (52%) patients displayed cHC-CCAs with stem cell features and 15 (48%) patients displayed the classical type. Two hundred and thirty-nine IHCCA patients were included in our study group, with 24 (10%) of them showing in the cholangiolocellular type. Nineteen EHBCA patients were included, all of them with perihilar Klatskin tumors. The diagnostic criteria for each group followed the guidelines established in the 2010 4th edition of the WHO classification of tumors of the digestive system [4]. A total of 913 patients were categorized into 8 disease groups based on their histological diagnosis and CK19 expression levels. The eight disease groups were: 1) CK19(−) HCC, 2) CK19(−) scirrhous HCC, 3) CK19(+) HCC, 4) cHC-CCA with stem cell features, 5) the classical type of cHC-CCA, 6) cholangiolocellular IHCCA, 7) non-cholangiolocellular IHCCA, and 8) EHBCA. The composition of the total cohort and disease groups is summarized in Fig. 1.

Clinical information, such as age, gender, presence of chronic liver disease, underlying etiology of chronic liver disease, post-operative tumor recurrence or metastasis and survival, preoperative treatment, and serum α-fetoprotein (AFP) was collected from existing medical records. Pathological information, such as tumor size, the number of tumors, vascular invasion, large vessel invasion, Edmondson-Steiner nuclear grade for HCCs, differentiation for CCAs, cellular type of tumor cells, histological pattern, the presence of desmoplastic stroma, and the pathological stage was collected from pathological reports and from reviewing the slides. Criteria for the determination of pT (pathologic T stage) followed the liver, intrahepatic bile duct tumor, or perihilar bile duct tumor staging guidelines established by the American Joint Committee on Cancer [19]. The clinicopathological parameters followed the general rules for the study of primary liver cancers [20]. This study was approved by the Institutional Review Board of Seoul National University Hospital (H-1011-046-339). Patient demographics can be seen in Table 1.

**Table 1:** Patient demographics (*N* = 913)

	HCC (624)	cHC-CCA (31)		IHCCA (239)		EHBCA (19)	*p*-value
		Stem cell (16)	Classical (15)	CLC(24)	Non-CLC(215)		
Sex (M:F ratio)	4.9	2.2	14	2	2.9	1.1	0.001*
Male	518(83)	11(69)	14(93)	16(67)	160(74)	10(53)	0.001*
Female	106(17)	5(31)	1(7)	8(33)	55(26)	9(47)	
Age (year, mean ± SD)	54 ± 10	51 ± 12	58 ± 8	60 ± 10	62 ± 9	58 ± 12	<0.001*
Chronic liver disease	593(95)	16(100)	12(80)	12(50)	39(18)	0(0)	<0.001*
Viral	557(94)	10(63)	9(75)	7(58)	27(69)	0(0)	<0.001*
Non-viral	36(6)	5(31)	3(25)	5(42)	12(31)	0(0)	
pT stage (AJCC 7th)							
pT1	256(41)	9(56)	3(20)	7(29)	81(38)	1(5)	0.01*
pT2-pT4	360(58)	7(44)	12(80)	17(71)	131(61)	17(89)	
Progress							
Recur or metastasis	395(63)	7(44)	10(67)	11(46)	144(67)	12(63)	<0.001*
PFS (median, month)	26	103	12	NA	11	20	<0.001*
Death							
Deceased	352(56)	6(38)	10(67)	6(25)	110(51)	18(95)	<0.001*
OS (median, month)	77	91	21	NA	61	18	<0.001*

*HCC* hepatocellular carcinoma, *CCA* cholangiocarcinoma, *IHCCA* intrahepatic cholangiocarcinoma, *EHBCA* extrahepatic bile duct carcinoma, *CLC* cholangiolocellular, *PFS* progression- free survival, *OS* overall survival, *NA* not applicable; *p-value <0.05

### Construction of tissue microarrays and immunohistochemical staining

**Fig. 2:**
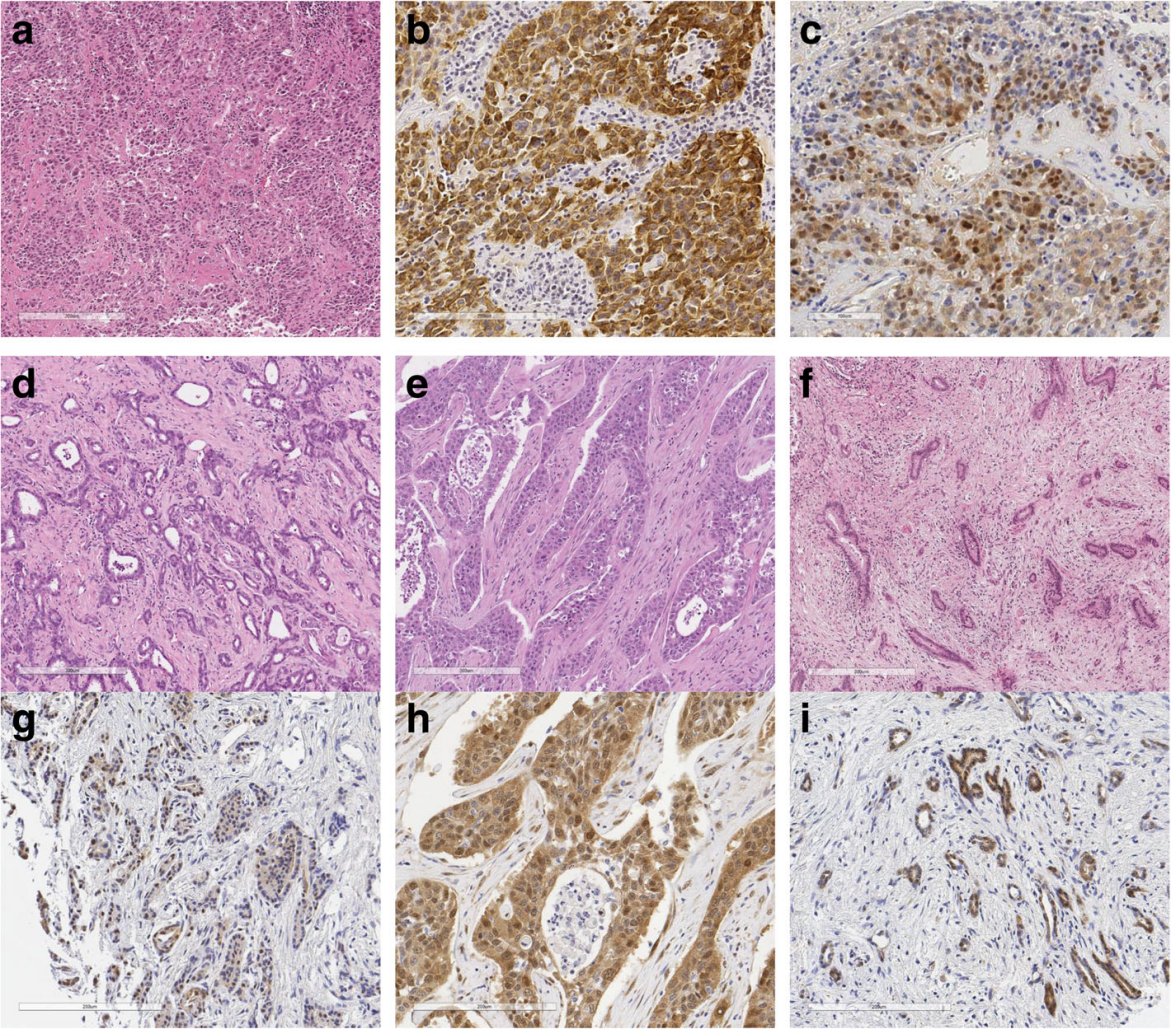
Representative sections and immunohistochemistry expression of HCC, cholangiolocellular IHCCA, non-cholangiolocellular IHCCA and EHBCA. **a** CK19(+) HCC, H&E (×200). **b** CK19 expression of CK19(+) HCC (×400). **c** YAP1 expression of CK19(+) HCC (×400). **d** Cholangiolocellular CCA, H&E (×200). **e** Non-cholangiolocellular IHCCA, H&E (×200). **f** EHBCA, H&E (×200). **g** YAP1 expression of cholangiolocellular IHCCA (×400). **h** YAP1 expression of noncholangiolocellular IHCCA (×400). **i** YAP1 expression of EHBCA (×400)

YAP1 expression was assessed on tissue microarrays. CK19 expression was assessed on a representative slide glass for 624 HCCs and 31cHC-CCAs. CK19(+) HCCs and cHC-CCA. In case of CK19(+) HCC or 31cHC-CCAs, CK19(+) tumor areas and CK19(−) tumor areas were selected for the tissue microarrays used for YAP1 staining. One core tissue specimen (2 mm in diameter) was collected from each individual paraffin-embedded tissue and rearranged in new tissue array blocks using a trephine apparatus (SuperBioChips Laboratories, Seoul, Korea). Each tissue microarray had four cores of normal liver, normal bile duct, and normal gastrointestinal tract mucosa as internal controls. Four-μm-thick glass slides were stained for YAP1 (mouse monoclonal anti-human YAP1, H-9, Cat.# sc-271,134, 1:100, Santa cruz biotechnology, Inc) and CK19 (mouse monoclonal anti-human cytokeratin 19, Clone RCK108, Cat. # M0888, 1:100, Dako) after an antigen retrieval process using Bond Epitope Retrieval Solution 2 at 99 °C for two minutes (Leica Biosystems, Wetzlar, Germany). The slides were automatically stained using Bond-Max IHC and ISH slide stainer and a Bond Polymer Refine Detection Kit (Leica Biosystems, Wetzlar, Germany). YAP1 expression was graded based on its nuclear expression. The intensity of nuclear staining was graded as negative, weak (1+), moderate (2+), or strong (3+). Antibody for CK19 was positively stained in cytoplasm and cellular membrane and the intensity of the positive staining was graded as negative, weak (1+), moderate (2+), or strong (3+). Two pathologists (KB Lee and KH Lee) evaluated the immunoreactivity of the samples and any discrepant cases were reevaluated. For the comparative analysis of protein expression and clinicopathological parameters, dichotomized values, as positive and negative, were used. The criteria for positivity was ≥2+ intensity in ≥5% of tumor cells (Fig. 2).

### Statistical analysis

Comparative analysis of clinicopathological parameters was conducted using the chi-squared (χ^2^) test or the Fisher’s exact test. Survival analysis was performed using Kaplan-Meier analysis and the Cox’s proportional hazards model. Progression-free survival (PFS) was defined as the time to local or distant progression. Overall survival (OS) was defined as the time to any cause of death. The results were considered statistically significant when *p-*values were <0.05. All tests were performed using the IBM SPSS version 21.

## Results

### Differences in clinicopathological features according to histological type

As summarized in Table 1, HCCs, cHC-CCAs, IHCCAs, and EHBCAs showed different clinical features. The male to female ratio was highly male-dominated in HCCs, lower in IHCCAs, and equally distributed in EHBCA patients. cHC-CCAs with stem cell features showed a similar sex ratio to IHCCA patients, but classical type cHC-CCAs demonstrated high male-dominance, much like HCCs. The mean age of patients was lower in HCCs and cHC-CCAs with stem cell features than in classical cHC-CCAs, IHCCAs, and EHBCAs. Association with chronic liver diseases was higher in HCCs and cHC-CCAs (ranging between 80% and 100%) than in IHCCAs or EHBCAs (21% or 0%). Interestingly, cholangiolocellular subtype IHCCAs showed a higher prevalence of chronic liver disease than non-cholangiolocellular IHCCAs (50% vs. 18%, *p*-value 0.001). The etiological factors of chronic liver disease tended to be more viral in the HCC group (94%) than in the other groups (58–79%). In spite of the different staging systems for histological subgroups, the rate of pT1, which is generally defined as a single mass limited in the liver without any vascular invasion, was the highest in cHC-CCAs with stem cell features and the lowest in EHBCAs (56% vs. 5% *p*-value 0.01). In terms of recurrence and metastasis, cHC-CCAs with stem cell features and cholangiolocellular IHCCAs had lower rates of disease progress when compared to classical cHC-CCAs, non-cholangiolocellular IHCCAs, and EHBCAs (46–50% vs. 11–27%, *p*-value <0.001). The rates of deceased patients were similar to the pattern seen in progress rates, as cHC-CCAs with stem cell features and cholangiolocellular IHCCAs had lower rates than the other types of hepatic carcinomas (25–31% vs. 51–95*%, p*-value < 0.001).

### Expression of YAP1 in 8 disease groups

Positive rates of YAP1 in the 8 disease groups are summarized in Table 2. Positive rates were the highest in the EHBCA (21%) group, followed by the CK19(+) HCC and non-cholangiolocellular IHCCA groups (10 and 11%). CK19(−) HCC, CK19(−) scirrhous HCCs, cHC-CCAs, and, cholangiolocellular IHCCAs showed low positive rates of YAP1 (0–5%). Disease groups with overt cholangiocytic differentiation (EHBCAs and non-cholangiolocellular IHCCAs) showed higher YAP1 expression levels than pure HCCs. However, disease groups with intermediate features showed heterogeneous results. The CK19(+) HCC group showed similar results to non-cholangiolocellular IHCCAs, but the remaining cHC-CCA and cholangiolocellular IHCCA groups displayed similar rates to the CK19(−) HCC group. Interestingly, one positive case in the cHC-CCA group was from a cHC-CCA with stem cell features that displayed a cholangiolocellular subtype. Representative pictures of positive YAP1 expression in each group can be seen in Fig. 2.

**Table 2:** Positive rates of YAP1 in 8 disease groups

	Number	Negative	Positive	*P-value*
CK19(−) HCC	491	482 (98)	9 (2)	<0.001*
CK19(−) scirrhous HCC	32	31 (97)	1 (3)	
CK19(+) HCC	101	91 (90)	10 (10)	
Stemness-feature Carcinoma	40	38 (95)	2 (5)	
Combined HC-CCA, stem cell feature	16	15 (94)	1 (6)	
Cholangiolocellular IHCCA	24	23 (96)	1 (4)	
Combined HC-CCA, classical type	15	15 (100)	0 (0)	
IHCCA, non-cholangiolocellular	215	192 (89)	23 (11)	
EHBCA	19	15 (79)	4 (21)	

*HCC* hepatocellular carcinoma, *CCA* cholangiocarcinoma, *IHCCA* intrahepatic cholangiocarcinoma, *EHBCA* extrahepatic bile duct carcinoma; *p-value <0.05

### Clinicopathological characteristics of HCCs and IHCCAs with positive YAP1 expression

The clinicopathological characteristics of HCCs with positive YAP1 expression are given in Table 3. YAP1 expressing HCCs had a higher ES nuclear grade and a higher serum AFP level (all *p*-value < 0.05). Non-trabecular compact patterns and desmoplastic stroma were more commonly identified in YAP1(+) HCCs (all *p*-value < 0.05). As previously mentioned, CK19(+) HCCs had high YAP1 positive rates ((−) vs. (+), 2% vs. 10%, *p*-value < 0.001). There was no difference in YAP1 immunoreactivity in terms of gender, age, tumor size, multiplicity, angioinvasion, large vessel invasion, pathologic T stage, preoperative treatment, the cell type of the tumor, histological pattern, and etiology of underlying chronic liver disease. Comparative analysis of clinicopathological characteristics of CCAs with positive YAP1 expression showed no significantly different parameters (Additional file 1: Table S1). YAP1(+) IHCCA seemed to show non-cholangiolocellular patterns, desmoplastic stroma, and non-mucinous type of IHCCA, but the distribution was too deviated to confirm the presence of a statistical difference.

**Table 3:** Clinicopathological features of hepatocellular carcinomas with positive YAP1 expression (*N* = 624)

		N (%)	*p*-value
Sex	*Male[518]* vs. *Female[106]*	16(3) vs. 4 (4)	*0.715*
Age (yr)	*≤55[324]* vs. *>55[300]*	9(3) vs. 11 (4)	*0.681*
Size (cm)	*≤5.0[379]* vs. *>5.0[235]*	12(3) vs. 8 (3)	*0.872*
Multiplicity	*Single[449]* vs. *Multiple[169]*	11(2) vs. 9 (5)	*0.072*
Vascular invasion	*Absent[330]* vs. *Present[286]*	10(3) vs. 10 (3)	*0.745*
Large vessel invasion	*Absent[578]* vs. *Present[37]*	18(3) vs. 2 (5)	*0.446*
pT stage (AJCC 7th)	*pT1–2[486]* vs. *pT3–4[138]*	16(3) vs. 4 (3)	*0.982*
ES nuclear grade	*1–2[300]* vs. *3–4[313]*	4(1) vs. 15 (5)	0.014*
Preoperative treatment	*Done[261]* vs. *Not done[315]*	13(5) vs. 6 (2)	*0.114*
Serum AFP (ng/ml)	*≤32[309]* vs. *>32[289]*	4(1) vs. 14 (5)	0.011*
Cell type	*Hepatic[539]* vs. *Non-hepatic[61]*	17(3) vs. 2 (3)	*0.958*
Histologic pattern	*Trabecular/acinar[487]* vs. *Compact[113]*	12(2) vs. 7 (6)	0.041*
Desmoplastic stroma	*Absent[462]* vs. *Present[113]*	9(2) vs. 9 (8)	<0.001*
Expression of CK19	*Negative[523]* vs. *Positive[101]*	10(2) vs. 10 (10)	<0.001*
Etiology of CLD	*Viral[557]* vs. *Non-viral[36]*	19(3) vs. 0 (0)	*0.621*

*p-value <0.05; ES nuclear grade, Edmonson-Steiner nuclear grade; *CLD* chronic liver disease

### Prognostic value of YAP1 in hepatocellular carcinomas and cholangiocarcinomas

**Fig. 3:**
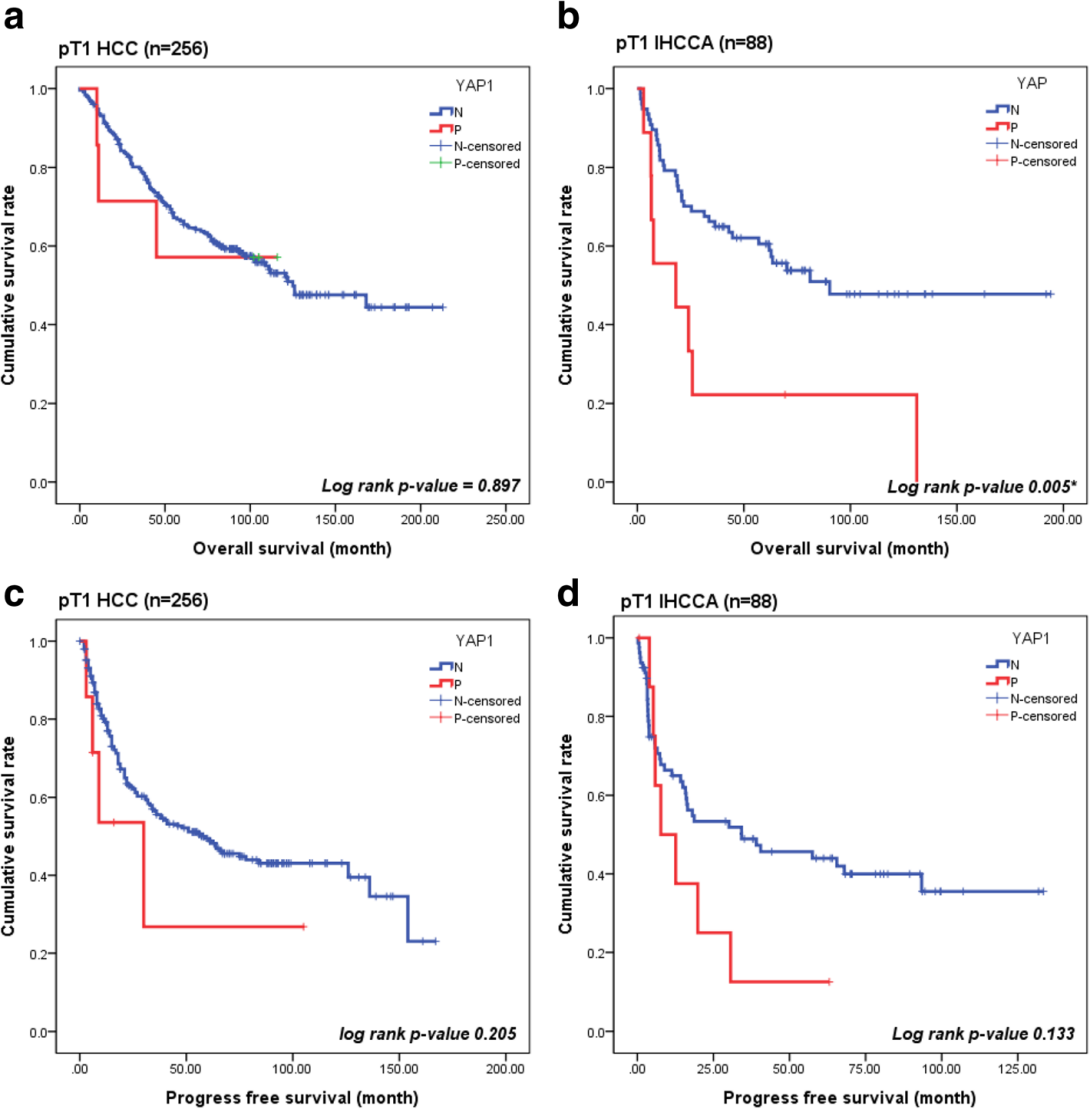
Kaplan-Meier curves for PFS and OS according to YAP1 expression. **a**, **c**, **d** Overall and progression free survival were no significant difference between two groups in pT1 HCCs and IHCCCs. **b** Overall survival in pT1 IHCCA was significantly longer in the YAP1 negative group than YAP1 positive group (*p*-value = 0.005*)

In order to adjust for the known prognostic factors, such as stage, differentiation, size, or vascular invasion, survival analysis was performed on pT1 stage cases. In the 256 pT1 HCC patients, YAP1(+) HCCs showed shorter PFS than YAP1(−) HCCs in the survival curve, but could not be identified as the statistically significant ((+) vs. (−), 39 ± 18 vs. 81 ± 5 months, mean ± SD, respectively, log rank *p*-value 0.205) (Fig. 3c). OS did not vary based on the YAP1 expression (Fig. 3a). In the 88 pT1 IHCCA cases, the OS of YAP1(+) IHCCAs was significantly lower than in YAP1(−) IHCCAs ((+) vs. (−) 39 ± 17 vs. 109 ± 10, log rank *p*-value 0.005) (Fig. 3b). The PFS of YAP1(+) IHCCAs was shorter than YAP1(−) IHCCAs, but, as in HCCs, could not be identified as statistically significant (18 ± 7 vs. 31 ± 7 months, log-rank *p*-value 0.133) (Fig. 3d).

## Discussion

Our study demonstrated that increased nuclear expression of YAP1 could be observed in cholangiocarcinomas (EHBCA and IHCCA) and a subset of hepatocellular carcinomas (CK19(+) HCC) and that nuclear YAP1 expression was correlated with poor overall survival in pT1 stage IHCCA patients. Interestingly, YAP1 expression rate in cHC-CCAs and cholangiolocellular IHCCAs was not so high as in EHBCAs, IHCCAs and CK19 (+) HCCs and was more similar to the expression levels seen in the CK19 (−) HCC group.

The Hippo pathway plays a major role in liver regeneration, development, and regulation. The determination of cellular lineage is of vital importance to this process. Activated YAP1 often induces hepatomegaly in animal models with mutated Hippo pathway genes, but the cellular components of the over-grown liver vary depending on the nature of the defective genes, e.g. hepatocyte proliferation, biliary hyperplasia, or abundant hepatic progenitor cells [21, 22] . Therefore, the determination of cell fate cannot be solely explained by activated YAP1. The level of activated YAP1 has also been associated with the determination of cell fates, differentiation to hepatocytes, and dedifferentiation to stem cells or progenitor cells [12]. In hepatic carcinogenesis, the genomic amplification of loci containing YAP1 in HCCs, suggests that YAP1 might possess an oncogenic effect [23]. As the determination of cell fate is extremely complicated, a simple inactivation of the Hippo pathway or the over-expression of YAP1 cannot induce liver cancer with an identical histological type [21]. Overexpression of YAP1 has been reported in the early phase of hepatocellular carcinomas, but the induction of hepatoblastoma required coactivation of β-catenin and interaction with PI3K has been reported to be involved in the induction of CCAs [24–26].

Based on this function of YAP1, we expected a gradual increase of YAP1 expression from HCCs to CCAs and culminating in high levels of overexpression in cHC-CCAs, cholangiolocellular CCAs, and CK19(+) HCCs, which are known to have “stemness” traits. However, only CK19(+) HCCs showed increased levels of YAP1 expression, as the levels of YAP1 expression in non-cholangiolocellular IHCCAs. Regardless of subtype, the cHC-CCAs showed a low positive rate of YAP1 expression, as did the CK19 (−) HCC group. The cholangiolocellular type of IHCCAs, which was assumed to originate from the hepatic progenitor cells in the canal of Hering, also had low YAP1 expression. The results we obtained were different from those seen in a previous study, which reported heightened levels of YAP1 in both HCCs with stemness and in cHC-CCAs when compared to HCCs without stemness [27]. As CK19(+) HCCs in our study were similarly defined as “HCCs with stemness”, increased YAP1 expression in CK19(+) HCCs was a consistent finding between two studies, but a similar result for cHC-CCAs could not be reproduced. CK19(+) expression in HCCs has been generally considered an indicator of stemness and poor prognosis for CK19(+) HCC patients was thought to be due to this stemness trait [10]. However, hepatic carcinomas with stemness features showed heterogeneous clinical behavior in our study cohort. Among the 8 disease groups, CK19(+) HCCs, cHC-CCAs with stem cell features, classical cHC-CCAs and cholangiolocellular type IHCCAs could be classified into stem cell feature carcinomas and this 4 disease groups could be divided into two different survival groups (log rank *p*-value = 0.001, Additional file 1: Figure S1). cHC-CCAs with stem cell features and cholangiolocellular IHCCAs were grouped with CK19(−) HCCs in displaying similar or better PFS than CK19(−) HCCs (median, 103 vs. 23 months, log rank *p*-value within groups 0.123 within groups) (Additional file 1: Fig. S1). However, CK19(+) HCCs were included in the poor prognosis group, together with IHCCAs and classical cHC-CCAs (median, 18 vs. 11 vs. 12 months, log-rank *p*-value within groups 0.670) (Additional file 1: Figure S1). Although other prognostic factors such as stages, preoperative or postoperative treatment modalities and difference of group size should be considered on the interpretation of prognosis, this result suggests that hepatic carcinoma with stemness features may be a heterogeneous entity and is consistent with the short mention about the conflict evidences of prognosis of cHA-CCA with stem cell feature [4]. Furthermore, hepatic carcinomas with stemness features are extremely heterogeneous in regards to their morphology and clinicopathological behavior as described in Table 1 and result section. Therefore, the expression of stemness markers in hepatic carcinomas might be due to poor differentiation or the acquisition of invasiveness by the tumor cells, and not due to the acquisition of stemness. Although we could identify an association between poor prognosis and activated YAP1 in hepatic carcinomas, this was only confirmed in the pT1 stage IHCCA patients. Nevertheless, we did observe a similar trend in the tumor progression of pT1 stage HCC patients. These results are consistent with previous studies reporting on the prognostic value of activated YAP1 in CCAs and HCCs [28, 29]

## Conclusions

Our study tried to elucidate the role of the Hippo pathway in the morphogenesis of the liver in regards to liver carcinomas and found that YAP1 activation was more commonly found in CCAs than pure HCCs. However, the heterogeneous pattern of YAP1 expression between cHC-CCAs and CK19(+) HCCs and the poor prognosis of YAP1 positive hepatic carcinomas suggests that YAP1 may have a preferential role in aggressive tumor behavior, rather than in the determination of cellular lineage in hepatic carcinomas.

## Additional file

Additional file 1: Table S1. Clinicopathological features of intrahepatic cholangiocarcinomas with positive YAP1 expression (*N* = 239). **Figure S1.** Progress free survival curve with disease groups. Stem cell feature carcinomas including stem cell feature of cHC-CCA and cholangiolocellular IHCCA had better PFS than CK19(−) HCC (median, 103 vs. 23 month, *p*-value 0.123). However, CK19(+) HCC was included poor prognosis group, together with IHCCA and classical cHC-CCA (median, 18 vs. 11 vs. 12 month, log rank *p*-value 0.670) (DOCX 318 kb).

## Abbreviations

AFP: α-fetoprotein
CCA: Cholangiocarcinoma
cHC-CCA: Combined hepatocellular-cholangiocarcinoma
CK19: Cytokeratin 19
EHBCA: Extrahepatic bile duct carcinoma
EpCAM: Epithelial cell adhesion molecule
HCC: Hepatocellular carcinoma
IHCCA: Intrahepatic cholangiocarcinoma
OS: Overall survival time
PFS: Progression-free survival time
YAP1: Yes-associated protein 1

## References

[1] Tung-Ping Poon R, Fan ST, Wong J (2000). Risk factors, prevention, and management of postoperative recurrence after resection of hepatocellular carcinoma. Ann Surg.

[2] Lee JI, Lee JW, Kim JM, Kim JK, Chung HJ, Kim YS (2012). Prognosis of hepatocellular carcinoma expressing cytokeratin 19: comparison with other liver cancers. World J Gastroenterol.

[3] Ghouri YA, Mian I, Blechacz B (2015). Cancer review: cholangiocarcinoma. J Carcinog.

[4] Theise ND, Nakashima O, Park YN, Nakanuma Y (2010). WHO classification of tumours of the digestive system.

[5] Tang KH, Ma S, Lee TK, Chan YP, Kwan PS, Tong CM (2012). CD133+ liver tumor-initiating cells promote tumor angiogenesis, growth, and self-renewal through neurotensin/interleukin-8/CXCL1 signaling. Hepatology.

[6] Yamashita T, Ji J, Budhu A, Forgues M, Yang W, Wang HY (2009). EpCAM-positive hepatocellular carcinoma cells are tumor-initiating cells with stem/progenitor cell features. Gastroenterology.

[7] Zamboni G KG, Hruban RH. WHO classification of tumours. Pathology and Genetics of Tumours of the Digestive System. 3^rd^ edn. Lyon: IARC Press; 2000.

[8] Aishima S, Oda Y (2015). Pathogenesis and classification of intrahepatic cholangiocarcinoma: different characters of perihilar large duct type versus peripheral small duct type. J Hepatobiliary Pancreat Sci.

[9] Chan AW, Tong JH, Chan SL, Lai PB, To KF (2014). Expression of stemness markers (CD133 and EpCAM) in prognostication of hepatocellular carcinoma. Histopathology.

[10] Kim H, Choi GH, Na DC, Ahn EY, Kim GI, Lee JE (2011). Human hepatocellular carcinomas with “Stemness”-related marker expression: keratin 19 expression and a poor prognosis. Hepatology.

[11] Zhu Q, Gurda G. YAP1 (yes-associated protein 1). Pancreapedia: The Exocrine Pancreas Knowledge Base 2014. http:// http://www.pancreapedia.org/molecules/yap1-yes-associated-protein-1. Accessed 15 Oct 2014.

[12] Yimlamai D, Christodoulou C, Galli GG, Yanger K, Pepe-Mooney B, Gurung B (2014). Hippo pathway activity influences liver cell fate. Cell.

[13] Nguyen Q, Anders RA, Alpini G, Bai H (2015). Yes-associated protein in the liver: regulation of hepatic development, repair, cell fate determination and tumorigenesis. Dig Liver Dis.

[14] Hu XB, Xin Y, Xiao YP, Zhao J (2014). Overexpression of YAP1 is correlated with progression, metastasis and poor prognosis in patients with gastric carcinoma. Pathology & Oncology Research.

[15] Li M, Lu J, Zhang F, Li H, Zhang B, Wu X (2014). Yes-associated protein 1 (YAP1) promotes human gallbladder tumor growth via activation of the AXL/MAPK pathway. Cancer Lett.

[16] Tschaharganeh DF, Chen X, Latzko P, Malz M, Gaida MM, Felix K (2013). Yes-associated protein up-regulates jagged-1 and activates the notch pathway in human hepatocellular carcinoma. Gastroenterology.

[17] Sun ZQ, Xu RW, Li XY, Ren WG, Ou CL, Wang QS (2015). Prognostic value of yes-associated protein 1 (YAP1) in various cancers: a meta-analysis. PLoS One.

[18] Barry ER, Morikawa T, Butler BL, Shrestha K, de la Rosa R, Yan KS (2013). Restriction of intestinal stem cell expansion and the regenerative response by YAP. Nature.

[19] Edge SB, Compton CC (2010). The American joint committee on cancer: the 7th edition of the AJCC cancer staging manual and the future of TNM. Ann Surg Oncol.

[20] Japan LCSGo. The general rules for the clinical and pathological study of primary liver cancer. Jpn J Surg 1989;19(1):98–129. 10.1007/BF02471576 2659865

[21] Yimlamai D, Fowl BH, Camargo F (2015). Emerging evidence on the role of the Hippo/YAP pathway in liver physiology and cancer. J Hepatol.

[22] Yimlamai D, Christodoulou C, Galli Giorgio G, Yanger K, Pepe-Mooney B, Gurung B (2014). Hippo pathway activity influences liver cell fate. Cell.

[23] Zender L, Spector MS, Xue W, Flemming P, Cordon-Cardo C, Silke J (2006). Identification and validation of oncogenes in liver cancer using an integrative oncogenomic approach. Cell.

[24] Li X, Tao J, Cigliano A, Sini M, Calderaro J, Azoulay D (2015). Co-activation of PIK3CA and yap promotes development of hepatocellular and cholangiocellular tumors in mouse and human liver. Oncotarget.

[25] Perra A, Kowalik MA, Ghiso E, Ledda-Columbano GM, Di Tommaso L, Angioni MM (2014). YAP activation is an early event and a potential therapeutic target in liver cancer development. J Hepatol.

[26] Tao J, Calvisi DF, Ranganathan S, Cigliano A, Zhou L, Singh S (2014). activation of β-catenin and Yap1 in human hepatoblastoma and induction of hepatocarcinogenesis in mice. Gastroenterology.

[27] Kim GJ, Kim H, Park YN (2013). Increased expression of yes-associated protein 1 in hepatocellular carcinoma with stemness and combined hepatocellular-cholangiocarcinoma. PLoS One.

[28] Li H, Wang SH, Wang GY, Zhang ZG, Wu XC, Zhang T (2014). Yes-associated protein expression is a predictive marker for recurrence of hepatocellular carcinoma after liver transplantation. Dig Surg.

[29] Pei T, Li Y, Wang J, Wang H, Liang Y, Shi H (2015). YAP is a critical oncogene in human cholangiocarcinoma. Oncotarget.

