# Supplementary material for: The correlation between poor prognosis and increased yes-associated protein 1 expression in keratin 19 expressing hepatocellular carcinomas and cholangiocarcinomas
Source: BMC Cancer. 2017 Jun 23;17:441. doi: 10.1186/s12885-017-3431-1 (PMC5481924; doi:10.1186/s12885-017-3431-1)
Supplement: Additional file 1: Table S1. — Clinicopathological features of intrahepatic cholangiocarcinomas with positive YAP1 expression (N = 239). Figure S1. Progress free survival curve with disease groups. Stem cell feature carcinomas including stem cell feature of cHC-CCA and cholangiolocellular IHCCA had better PFS than CK19(−) HCC (median, 103 vs. 23 month, p-value 0.123). However, CK19(+) HCC was included poor prognosis group, together with IHCCA and classical cHC-CCA (median, 18 vs. 11 vs. 12 month, log rank p-value 0.670) (DOCX 318 kb). [file 12885_2017_3431_MOESM1_ESM.docx]

Additional file 1

| Table S1. Clinicopathological features of intrahepatic cholangiocarcinomas with positive YAP1 expression (N=239) | | | |
| --- | --- | --- | --- |
|  |  | N (%) | *p-value* |
| Sex | *Male[176] vs. Female[58]* | 19(11) vs. 5 (9) | *0.63* |
| Age (yr) | *≤55[58] vs. >55[179]* | 7(12) vs. 17 (9) | *0.573* |
| Size (cm) | *≤5.0[128] vs. >5.0[104]* | 13(10) vs. 9 (9) | *0.823* |
| pT stage (AJCC 7th) | *pT1-2[155] vs. pT3-4[81]* | 18(12) vs. 6 (7) | *0.37* |
| pN stage (AJCC 7th) | *pN0[106] vs. pN1[63]* | 14(13) vs. 7 (11) | *0.69* |
| Lymphatic invasion | *Absent[147] vs. Present[84]* | 15(10) vs. 8 (10) | *0.868* |
| Vascular invasion | *Absent[143] vs. Present[83]* | 16(11) vs. 5 (6) | *0.24* |
| Perinueral invasion | *Absent[157] vs. Present[72]* | 16(10) vs. 7 (10) | *0.913* |
| Differentiation | *well-moderate[166] vs. poor[73]* | 19(11) vs. 5 (7) | *0.553* |
| Cell type | *Intestinal[43] vs. Nonintestinal[196]* | 6(14) vs. 18 (9) | *0.346* |
| Histologic pattern | *Tubular[202] vs. Nontubular[35]* | 19(9) vs. 5 (14) | *0.592* |
| Cholangiolocellular type | *Absent[215] vs. Present[24]* | 23(11) vs. 1 (4) | *0.483* |
| Desmoplastic stroma | *Absent[37] vs. Present[201]* | 1(3) vs. 23 (11) | *0.139* |
| Overproduction of mucin | *Absent[216] vs. Present[17]* | 23(11) vs. 0 (0) | *0.387* |
| Association with CLD | *Absent[188] vs. Present[51]* | 22(12) vs. 2 (4) | *0.12* |
| * p-value < 0.05; CLD, chronic liver disease | |  |  |

**Figure S1. Progress free survival curve with disease groups.** Stem cell feature carcinomas including stem cell feature of cHC-CCA and cholangiolocellular IHCCA had better PFS than CK19(-) HCC (median, 103 vs. 23 month, p-value 0.123). However, CK19(+) HCC was included poor prognosis group, together with IHCCA and classical cHC-CCA (median, 18 vs. 11 vs. 12 month, log rank p-value 0.670).

**
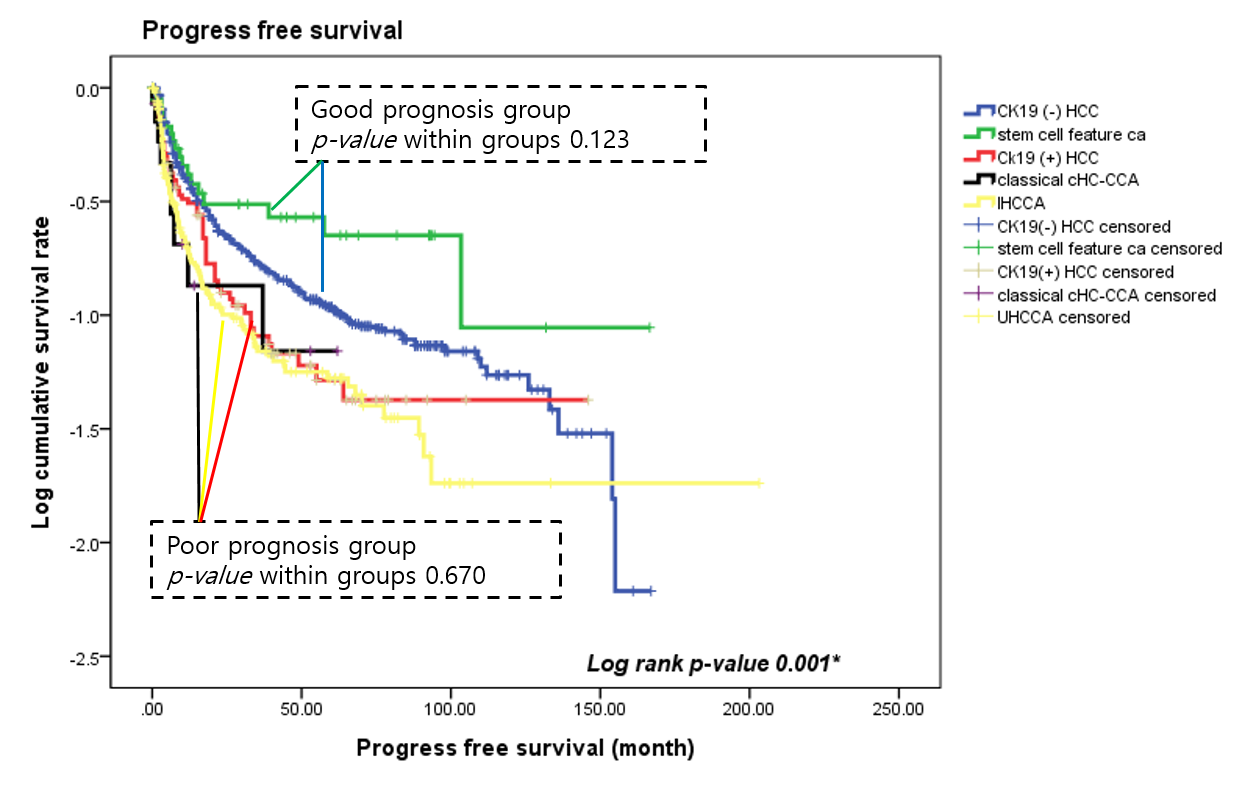
**
